# Advances in ATR-FTIR Spectroscopic Imaging for the Analysis of Tablet Dissolution and Drug Release

**DOI:** 10.3390/molecules28124705

**Published:** 2023-06-12

**Authors:** Céline van Haaren, Marieke De Bock, Sergei G. Kazarian

**Affiliations:** Department of Chemical Engineering, Imperial College London, London SW7 2AZ, UK; c.van-haaren21@imperial.ac.uk (C.v.H.);

**Keywords:** FTIR spectroscopic imaging, chemical imaging, dissolution, drug release, solid oral dosage forms

## Abstract

One of the major challenges in the development of effective pharmaceutical formulations for oral administration is the poor solubility of active pharmaceutical ingredients. For this reason, the dissolution process and drug release from solid oral dosage forms, such as tablets, is usually thoroughly studied in order to understand the dissolution behaviour under various conditions and optimize the formulation accordingly. Standard dissolution tests used in the pharmaceutical industry provide information on the amount of drug released over time; however, these do not allow for a detailed analysis of the underlying chemical and physical mechanisms of tablet dissolution. FTIR spectroscopic imaging, by contrast, does offer the ability to study these processes with high spatial and chemical specificity. As such, the method allows us to see the chemical and physical processes which occur inside the tablet as it dissolves. In this review, the power of ATR-FTIR spectroscopic imaging is demonstrated by presenting a number of successful applications of this chemical imaging technique to dissolution and drug release studies for a range of different pharmaceutical formulations and study conditions. Understanding these processes is essential for the development of effective oral dosage forms and optimization of pharmaceutical formulations.

## 1. Introduction

Attenuated total reflection-Fourier transform infrared (ATR-FTIR) spectroscopic imaging is a powerful chemical imaging technique which is used to study a variety of materials and processes. It enables label-free and non-destructive characterisation of a broad range of samples and can provide insight into the distribution of components as well as intermolecular interactions in the studied sample. The capabilities of so-called “chemical photography” for drug dissolution studies were demonstrated about 20 years ago, when the crystallisation of ibuprofen during dissolution was spectroscopically imaged for the first time in the authors’ laboratory using macro-ATR FTIR spectroscopic imaging [1]. Further developments in the field of vibrational spectroscopy and spectroscopic imaging have led to the extensive use of advanced spectroscopic techniques in the pharmaceutical industry, particularly in research on the dissolution and drug release of pharmaceutical formulations [2,3]. A large amount of useful information can be collected rapidly and easily in a single measurement, as every FTIR image, besides providing spatial information, contains the spectral data of each individual component in the sample. FTIR spectroscopic imaging therefore has a great advantage over other analytical tools when it comes to studying multi-component and dynamic systems, making it a suitable technique for studying tablet dissolution and drug release [4]. Because of its high chemical specificity and spatial resolution, it can be employed as a stand-alone technique with the capability to show “a full picture” of the sample under study [5]. However, it can be complemented by other chemical imaging techniques such as Raman spectroscopic mapping, magnetic resonance imaging (MRI) or ultraviolet (UV) imaging [6].

There are several ways to administer a pharmaceutical drug to a patient; however, oral administration remains the preferred route for most formulations, as it is non-invasive, cost-effective and convenient in use. Despite these advantages, numerous oral medications display suboptimal dissolution and poor absorption [7]. Tablets are the most common form of oral drug delivery. Drug release from tablets is influenced by several factors, including the physicochemical properties of the tablet components, the ratio of the tablet components and the environmental conditions during dissolution. In order for the active pharmaceutical ingredient (API) to be absorbed by the blood and have a therapeutic effect, effective dissolution of the tablet is crucial. For this reason, tablet dissolution is thoroughly studied for various formulations and conditions [8,9]. FTIR spectroscopic imaging has proved to be particularly useful for tablet dissolution studies, as the results may provide insight into the dissolution behaviour and distribution of components within the dosage form as well as water ingress, swelling and interactions with excipients [2].

The purpose of this article is to provide an overview of the recent developments in understanding and improving tablet dissolution through the use of FTIR spectroscopic imaging. Firstly, tablet dissolution and its challenges will be discussed. Then, a brief background on FTIR spectroscopy will be provided, followed by a more detailed discussion of ATR-FTIR spectroscopic imaging. Finally, the findings of several studies focused on improving tablet dissolution and drug release will be presented, which include a range of tablet formulations and dissolution conditions.

## 2. Tablet Dissolution and Reoccurring Problems in Drug Release

### 2.1. Tablet Formulation

Many therapeutic compounds are poorly soluble in water, posing a major challenge for formulation scientists. Therefore, the addition of an excipient that allows for enhanced aqueous solubility and permeability is often necessary in the formulation of an effective dosage form. Excipients are pharmaceutically inactive substances which may be added for various reasons, such as improving drug stability or drug uniformity. Furthermore, they may be used to improve manufacturability (e.g., fillers, lubricants, glidants), improve disintegration (e.g., disintegrants) or simply improve the taste and appearance of the dosage form (e.g., sweeteners, flavourings, additives and colourants). Most importantly, however, excipients are added to increase the solubility and/or absorption of a drug, thereby improving the bioavailability of the API in the body. Commonly used excipients include salts, sugars, saccharides, proteins and polymeric materials [8,10,11]. In addition to adding excipients to the oral dosage form, there are several other strategies to improve the poor solubility of drugs and thereby enhance their dissolution rate. These include particle size reduction, co-crystallisation with a coformer, the use of cosolvents, formation of solid dispersions and formation of inclusion complexes [12,13,14,15,16,17].

### 2.2. Tablet Dissolution and Drug Release

Conventional dosage forms typically result in a rapid increase in drug level in the blood, followed by a decrease over time while the drug is metabolized and cleared from the body. These immediate release formulations often require the administration of multiple doses at intervals to achieve the minimum effective level of drug for a sufficient period of time while keeping the drug concentration below the minimum toxic concentration. Controlled release dosage forms allow for a more effective drug release, as they aim to reduce redosing by maintaining the drug level for a longer period of time and exhibiting a controlled release rate. Generally, a controlled release system is characterized by a period of fast release after which drug release follows first-order kinetics, i.e., the drug release rate is proportional to the amount of drug in the system. These first-order systems can achieve prolonged drug release over hours, days, weeks or even months. In some cases, zero-order release systems may be desirable, which can achieve constant release rates for a prolonged period of time [18,19].

**Figure 1:**
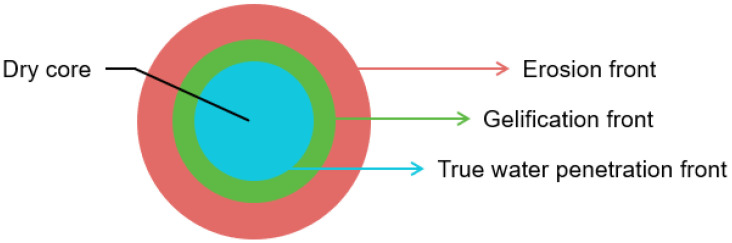
Schematic of tablet dissolution in a monolithic matrix system.

There are several mechanisms through which controlled drug release can be achieved. One of the most common controlled release systems in the case of oral dosage forms is a monolithic matrix system in which the drug is dispersed in a polymeric matrix. Release of the drug may be governed by diffusion, swelling and/or erosion of the matrix as a result of water penetration, depending on the design of the dosage form [20]. Furthermore, drug release from the dosage form may be chemically controlled, i.e., exposure to specific environmental conditions causes erosion of the dosage form and subsequent drug release [21,22]. Dissolution of swellable monolithic matrix systems has been studied through various imaging methods, including visible photography, fluorescence imaging, ATR- and transmission-FTIR imaging, scanning electron microscopy and magnetic resonance imaging [23]. Figure 1 presents the dissolution of a swellable monolithic matrix system as studied by ATR-FTIR spectroscopic imaging [24]. Here, dissolution is characterised by three concentric fronts. The innermost front is attributed to the true water penetration front, where water enters the porous tablet but minimal gel formation takes place. The second front is attributed to gelification, i.e., the transition from a glassy polymer to a gel-layer. Lastly, the outermost front can be described as the erosion or dissolution front, where the water content is high and the matrix disappears [24,25]. The behaviour of the different fronts during tablet dissolution and particularly the thickness of the gel layer are indicative of the drug release kinetics and are therefore worth studying [26].

Standard United States Pharmacopeia (USP) dissolution tests only provide information on the drug release over time, but give no insight into the distribution of components in the tablet or dynamics of tablet dissolution. For this purpose, chemical imaging techniques may be employed. ATR-FTIR spectroscopic imaging is able to characterize a sample with high chemical specificity and high spatial resolution, providing much more and more relevant information on tablet dissolution behaviour compared to the USP dissolution test by itself. If desired, the flow cell setup that is generally used for ATR-FTIR spectroscopic imaging can be coupled to a UV-vis spectrophotometer, which allows for the simultaneous measurement of total released drug during the flow experiment [6,27]. Furthermore, since FTIR spectroscopic imaging does not require complex sample preparation or labelling, it is possible to study a dosage form under various different conditions, e.g., by selecting temperature, buffer pH, ionic strength and flow rate, which is particularly useful for formulation optimization.

## 3. FTIR Spectroscopy

FTIR spectroscopy is a technique that provides information on the molecular structure of a sample based on the absorption of infrared light (IR) by the sample. Molecules will only be IR-active if their molecular vibrations constitute a change in the dipole moment. In those cases, light will be absorbed at specific frequencies, i.e., the frequencies matching the vibrational frequencies. The resulting absorption spectrum is highly dependent on the molecular structure and functional groups present in the sample, and will therefore provide a “fingerprint” for the measured material. This is what makes FTIR spectroscopy a powerful characterisation technique with high chemical specificity [28]. In the mid-infrared region (4000–400 cm^−1^), most of the vibrations are bending and stretching vibrations. Strong absorption is detected for molecules with a permanent dipole, such as carbonyl groups. Stretching bond frequencies depend on bond strength. Interaction with other groups, as hydrogen bonding, which influence the bond strength will be recognized in the spectrum by a frequency shift. Stretching vibrations, such as S-H, O-H, N-H and C-H, are generally detected at higher wavenumber (3500–2550 cm^−1^). Lower wavenumbers are typically assigned to carbon skeleton fingerprint and bending vibrations [29,30,31,32,33].

### 3.1. Modes

Vibrational spectroscopic analysis can be carried out via three main modes: transmission, external reflection and attenuated total reflection (ATR). Depending on the application, sample form and size, experimental setup and/or research objectives, the most suitable mode of FTIR spectroscopy is selected.

#### 3.1.1. Transmission

In transmission mode, incident radiation passes directly through the sample, where infrared light is absorbed at specific wavelengths. The light that is not absorbed is transmitted onto a detector, resulting in an absorption spectrum. This mode is widely adopted because of its straightforward application, availability of equipment, and excellent signal-to-noise ratio. However, one important condition is that the sample must be very thin, typically 5–50 µm, in order for the infrared radiation to pass through the sample and not be completely absorbed [4,34]. This complicates sample preparation and makes it impossible to investigate commercial tablets, as these are much greater in size. Furthermore, the strong absorbance of IR radiation by water can interfere with the spectral bands of the studied sample, making spectral analysis challenging [2]. For these reasons, transmission mode may not be the most suitable option for studying tablet dissolution using FTIR spectroscopy.

#### 3.1.2. External Reflection

When using external reflection spectroscopy, the intensity of radiation that is reflected back by the surface of a sample is measured over a range of wavelengths at the desired angle. This can be particularly useful when studying the surface of a sample, for example, in thin films. As opposed to transmission mode, external reflection requires minimal sample preparation. In addition, it is a completely contactless measurement technique [35,36].

#### 3.1.3. Attenuated Total Reflection

**Figure 2:**
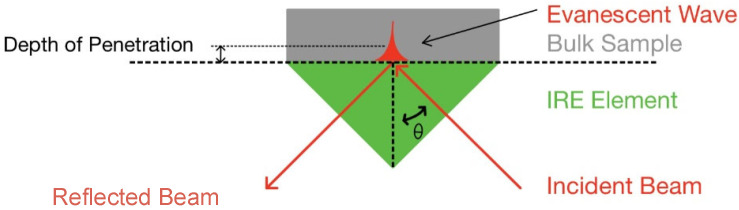
Schematic of attenuated total reflection at IRE surface.

In attenuated total reflection mode, infrared light is reflected once or multiple times within the internal reflection element (IRE) before being directed to the detector. For total internal reflection to take place, the IR light needs to enter the IRE at an angle greater than the critical angle and the IRE material must have a higher refractive index than the sample. At the interface between the IRE and the sample, an evanescent wave is generated which is able to penetrate the sample when it is in close contact with the IRE, as shown in Figure 2. The evanescent wave usually only penetrates a few micrometres into the sample, thereby allowing samples of any size to be measured [37].

The depth of penetration is defined as the distance from the IRE surface at which the electric field has decreased to e^−1^ of its value at the surface and can be calculated using Equation (1). Here, λ is the wavelength of the IR radiation, θ is the angle of incidence and $n_{\mathrm{sample}}$ and $n_{\mathrm{IRE}}$ are the refractive indices of the sample and crystal, respectively [38].

(1) $$d_{p}=\frac{\lambda }{2{\pi n}_{\mathrm{IRE}}\sqrt{\sin^{2}\theta -{\left(\frac{n_{\mathrm{sample}}}{n_{\mathrm{IRE}}}\right)}^{2}}}$$

ATR-FTIR spectroscopy allows for the analysis of a range of sample forms, including solid samples, solutions and hydrated films, requiring minimal sample preparation. In ATR mode, sample thickness does not have to be controlled as opposed to the samples measured in transmission, making it a suitable method for studying tablet dissolution. However, in order to obtain high-quality spectra, sufficient contact between the sample and the IRE is required [37,39].

One disadvantage of measuring samples in ATR mode is that in order to obtain an accurate absorption spectrum that is comparable to the absorption spectrum obtained through transmission measurement, the optical constants n and k, the real and imaginary refractive indices, need to be determined. This can be carried out by measuring the material at different angles of incidence; however, as a typical ATR accessory does not allows for this, a more commonly used approach is the Kramers–Kronig (K-K) method, which is based on K-K transformation, where the real part (n(ν)) can be found from the imaginary part (k(ν)) and vice versa using integral relations, and on the implementation of Fresnel’s equations [40,41].

### 3.2. Quantification of IR Absorption Spectra

To quantitatively analyse data in IR spectroscopy, application of the Beer–Lambert law is essential. The Beer–Lambert law states that the relationship between the measured absorbance (A) and the concentration (c), molar absorptivity (ε) and optical path length (l) is linear, as shown in Equation (2). This allows for the quantification of a specific component based on the absorption spectrum.

(2) A=εcl

However, it is important to note that the Beer–Lambert law only holds for a range of absorbance values and non-linearity occurs at high absorbance and at very low absorbance. In this case, quantification becomes imprecise. For measurements in transmission mode, controlling the thickness of a sample is particularly important to allow for quantitative analysis. In ATR-FTIR spectroscopy, off-scale absorbance measurements can be easily avoided by controlling the depth of penetration through the selection of a suitable IRE and angle of incidence [29,42,43].

## 4. ATR-FTIR Spectroscopic Imaging for Tablet Dissolution Studies

### 4.1. FTIR Spectroscopic Imaging

In FTIR spectroscopic imaging, an FTIR spectrometer is coupled with a focal plane array (FPA) detector, allowing for the collection of multiple spectra from different locations simultaneously. FPA detectors have 32 × 32 pixels, 64 × 64 pixels or 128 × 128 pixels with pixel dimensions of 40 µm × 40 µm. They are cooled with liquid nitrogen and can usually detect a spectral range of 4000–900 cm^−1^ [44]. Each pixel contains an absorption spectrum with chemical information on the sample in that specific location. By integrating over a band in the absorption spectrum that is characteristic for the component of interest and plotting the value for each pixel, a chemical image can be generated. Compared to FTIR mapping, where an image is generated based on point-by-point measurements of the sample using a single-element detector, FTIR imaging provides much shorter measurement times [44,45,46].

FTIR spectroscopic imaging has countless applications in the medical and biochemical fields. Promising developments have been made in cancer biopsies which can enable early diagnosis or a better understanding of the distribution of exogenous molecules [47,48]. It has been used to image proteins, skin and arteries to detect and understand different medical conditions [49,50]. Furthermore, FTIR imaging is applied in pharmaceutical drug release [1,4,5,34], microfluidics [51,52], membrane science [53], biopharmaceutical research [54,55] and artwork conservation [56,57]. In polymeric materials, spectroscopic imaging is performed to study the degradation behaviour, identify the distribution of components and visualise inter- or intramolecular interactions in polymer blends [58,59]. This technique has also proven its effectiveness in forensic science as extensive research has been carried out regarding the chemical characterisation of latent fingerprints [60,61].

### 4.2. ATR-FTIR Spectroscopic Imaging for Dissolution Studies

As mentioned previously, attenuated total reflection is the most suitable mode for tablet dissolution studies, as sample thickness is not a limiting factor. ATR-FTIR spectroscopic imaging requires an ATR accessory which consists of an internal reflection element (IRE) and internal optics to direct the IR beam. Several factors influence the choice of ATR accessory: throughput, refractive index, crystal material hardness and pH tolerance, and spectral range. Furthermore, size and the interaction with the sample should be considered. Zinc selenide (ZnSe) and diamond are the two most widely used materials for ATR measurements. Whilst ZnSe offers a larger surface area and is relatively inexpensive, it is not recommended when working in a pH range outside of 5–9 and scratches easily. Diamond, on the other hand, is a good choice in a wide pH range, withstanding high pressure and temperatures whilst also offering the highest refractive index. However, the material is expensive and the most commonly used accessories with diamond IRE therefore have a relatively small surface area. Furthermore, germanium (Ge) may be used, which has a notably higher refractive index and results in a four times improvement in spatial resolution when used with the microscope objective. It is generally used for micro-ATR-FTIR imaging and carbon-filled samples. Silicon (Si) is recommended for measurements in the far-IR spectral region (400–10 cm^−1^) [62]. An overview of IRE properties is presented in Table 1.

**Figure 3:**
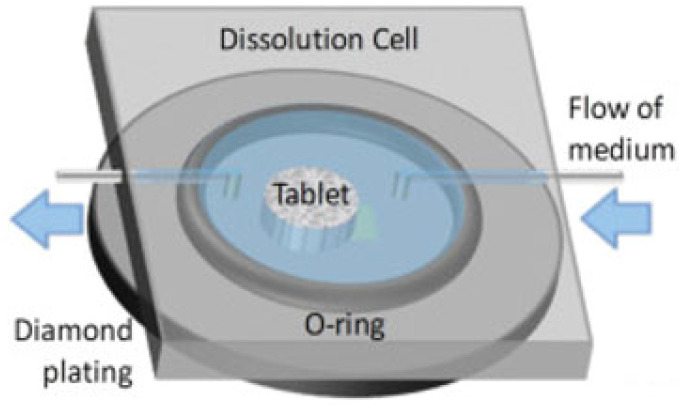
In situ tablet dissolution setup on diamond IRE. Adapted with permission [64].

In a typical experimental setup for a tablet dissolution study, the sample is placed onto the IRE, surrounded by a flexible O-ring and secured using a top plate. The sample will be sandwiched between the IRE and top plate with sufficient pressure to (i) seal the flow cell to prevent leakage of the medium and (ii) provide good contact between the sample and crystal surface. Depending on the imaging area and the size of the sample, measurements are taken of either the whole tablet or only part of the tablet, typically including the interface between the sample and medium [24,63,64]. An overview of the tablet dissolution setup is shown in Figure 3.

FTIR spectroscopic imaging experiments typically produce very large datasets, particularly in the case of dissolution studies where measurements are taken over a series of time points. Each measurement creates a three dimensional data cube of the array of pixels, x by y, times the number of wavenumbers over which IR absorption is measured. Before the data can be analysed, pre-processing is often necessary, which may include baseline correction, normalisation and de-noising or smoothing [44,45]. Depending on the compound of interest and its absorption peak in the resulting spectra, univariate data analysis may be sufficient to answer the research question. For tablet dissolution studies, mapping specific components within the sample and following the distribution of these components over time is one of the main approaches. Furthermore, average spectra from areas of interest may be extracted and compared over time. In some cases, multivariate data analysis methods are necessary to obtain the desired information [45].

### 4.3. Complementary Chemical Imaging Techniques

Besides FTIR spectroscopic imaging, there are several other imaging techniques which may provide useful insights into the dissolution behaviour of the studied sample. These include magnetic resonance imaging (MRI), Raman imaging and UV imaging. Each of these imaging techniques relies on different principles and may therefore reveal different chemical information and/or achieve different spatial and temporal resolution. Combining two or more of these analytical tools will result in a more thorough understanding of the sample and its dissolution characteristics [6].

#### 4.3.1. MRI

Magnetic resonance imaging is a non-invasive imaging technique which can provide insights into the hydration of a sample during dissolution. The imaging technique is based on the phenomenon of nuclear magnetic resonance, in which atomic nuclei, typically hydrogen nuclei, are magnetized by a static magnetic field and exposed to radio-frequency radiation of a specific resonance frequency, generating a signal [65]. In tablet dissolution studies, MRI generally maps the water concentration and mobility, offering the possibility to quantify tablet dimensions as a result of hydration and the potential effects of hydration, including water penetration, gel formation, swelling and/or erosion. Moreover, interactions between protons and the matrix system can be observed as well as the free water and water bound to macromolecules, such as polymer chains [66]. Although hydrogen nuclei are most commonly imaged through MRI in tablet dissolution studies, it is possible to image other nuclei, for example, ^19^F nuclei [67]. The spatial resolution of the pixels obtained through MRI is typically in the range of 200–500 µm [67,68]. One drawback for pharmaceutical research is that, in general, MRI is mainly used to measure liquids rather than solids. Imaging of solid materials is possible; however, this comes with S/N issues and long acquisition times [66,69]. Alternative approaches have been explored to study the solid samples indirectly, for example, by filling the cavities and voids of a tablet with oil-based fluid to obtain the tablet density or porosity distribution [70].

#### 4.3.2. Raman Mapping

Raman mapping is an imaging technique that, similar to FTIR spectroscopic imaging, makes use of the molecular vibrations in molecules to obtain information on the chemical structure of a sample. Raman spectroscopy relies on the Raman effect, where photons interacting with the sample either gain energy (anti-Stokes scattering) or lose energy (Stokes scattering), resulting in their scattering at a shorter or longer wavelength, respectively. For pharmaceutical samples, Raman spectroscopy typically involves Stokes scattering, where the molecules in the sample undergo an increase in vibrational energy and the scattered photons are red-shifted. While FTIR spectroscopy is dependent on a change in dipole moment during vibration, Raman spectroscopy is dependent on a change in polarizability. Because of this, water bands will appear strongly in the IR absorption spectrum, but only weakly in the Raman spectrum [71]. In Raman mapping, an image is generated by moving the sample into the laser focus and measuring an array of sample points. This can be carried out in point-by-point fashion, or, to speed up the process, a line can be illuminated rather than a single point. Alternatively, Raman imaging can be employed, where the spectral intensity of the total area of interest is measured at once. However, using this method, only a discrete part of the full spectral range, i.e., a slice of wavenumbers, is recorded at one moment rather than the entire spectrum. For this purpose, optical filters may be used. With this setup no sample movement is possible during the experiment, ruling out the opportunity to study dynamic systems over time [2,71]. Raman mapping may be particularly useful for studying the distribution and particle size of specific components within a dosage form [72,73]. Furthermore, it can be used to monitor structural changes at the free tablet surface during dissolution, such as crystallisation [6].

#### 4.3.3. UV-Vis Imaging

UV imaging is based on the absorption of light in the visible and UV range by the chromophores of molecules in the sample under study. Conventionally, UV-vis spectrophotometry is used in the pharmaceutical industry for the quantification of chemical substances based on the Beer–Lambert law. However, by applying UV imaging, where spatially resolved absorbance maps are generated using a phosphor-coated complementary metal oxide semiconductor (CMOS) camera chip, drug dissolution processes such as diffusion, swelling, crystal growth, precipitation and partitioning phenomena can be studied in real-time [74,75,76]. Since most UV-vis instruments generally only use the light of a single wavelength, it can be challenging to distinguish the absorption by the dissolved drug from scattering or obscuration of particles and solid matter. This is particularly difficult in complex systems containing multiple excipients. Dual wavelength instrumentation, which has been developed in recent years, provides more opportunities for UV imaging to be used as an imaging technique for drug dissolution studies, including the functional characterisation of excipients in solid oral dosage forms [77].

## 5. Advances in FTIR Spectroscopic Imaging for Tablet Dissolution and Drug Release

### 5.1. Drug Formulation and Fabrication

Since many of the newly developed therapeutic compounds are poorly soluble in aqueous environments, formulation scientists are challenged to come up with tablet formulation and fabrication strategies that will improve solubility and drug release rate. One of these strategies is creating amorphous solid dispersions (ASDs), which contain the drug in an amorphous form dispersed with a polymer to reduce or prevent drug crystallisation. Several fabrication methods have been developed to achieve the creation of the amorphous form of a drug, including lyophilisation, spray-drying, hot-melt extrusion and cryo-milling, all aiming to increase drug solubility and improve dissolution from the dosage form [78].

**Figure 4:**
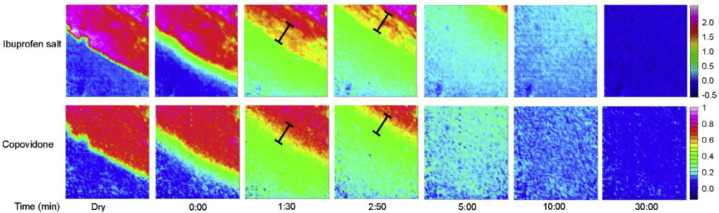
ATR-FTIR images showing the dissolution of ibuprofen salt and copovidone polymer extrudate. Chemical images were obtained by integrating over the range 1573–1530 cm^−1^ for ibuprofen salt and 1044–1013 cm^−1^ for copovidone. Image size: 0.58 × 0.64 mm^2^. Reprinted with permission [79].

**Figure 5:**
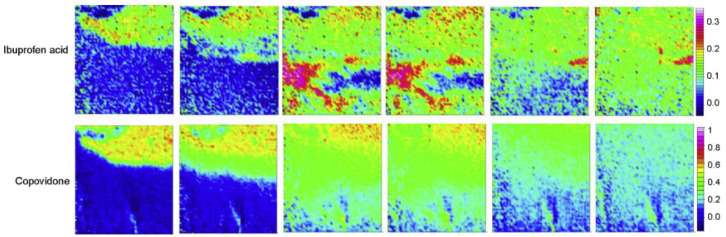
ATR-FTIR images showing the dissolution of ibuprofen acid and copovidone polymer extrudate. Chemical images were obtained by integrating over the range 1523–1501 cm^−1^ for ibuprofen acid and 1044–1013 cm^−1^ for copovidone. Reprinted with permission [79].

A recent collaborative industrial study by Pudlas et al. investigated the role of drug–polymer interactions in amorphous solid dispersion (ASD) formulations produced through hot-melt extrusion [79]. Two different hydrophilic polymers were compared, i.e., copovidone and Soluplus^®^, and two forms of ibuprofen, i.e., salt and acid form. Additionally, sodium carbonate was added to part of the formulations to study the effect of a pH-modifying component on drug release. The dissolution behaviour of all formulations was analysed in a flow cell via macro ATR-FTIR spectroscopic imaging and the total amount of drug released was measured using an in-line UV-vis spectrometer. For comparison, all formulations were analysed using a conventional dissolution test for which a USP apparatus 2 was employed. When comparing the dissolution behaviour of the ibuprofen acid formulation with the ibuprofen salt formulation, it was found that the ibuprofen salt extrudate (Figure 4) dissolved a lot quicker than the ibuprofen acid extrudate (Figure 5). By analysing the spectra extracted from ATR-FTIR chemical images, it was possible to investigate the underlying reason for this. It was found that ibuprofen salt does not interact with either of the polymers, whereas the ibuprofen acid does interact with both copovidone and Soluplus^®^ through hydrogen bonds. These drug–polymer interactions are happening on the hydrophilic part of the polymer and prevent water from interacting with the polymer. Consequently, a significantly slower drug release was observed for the ibuprofen acid extrudate. Furthermore, it was found that addition of sodium carbonate resulted in reduced drug–polymer interactions and therefore led to increased drug release for both polymers. This was attributed to the hydroxyl group separating from ibuprofen and the formation of the sodium salt form of the drug, which exhibits higher solubility. Lastly, the extrudates containing copovidone were found to dissolve faster than those containing Soluplus^®^, which was explained by the higher hydrophilicity of copovidone compared to Soluplus^®^ [79].

**Figure 6:**
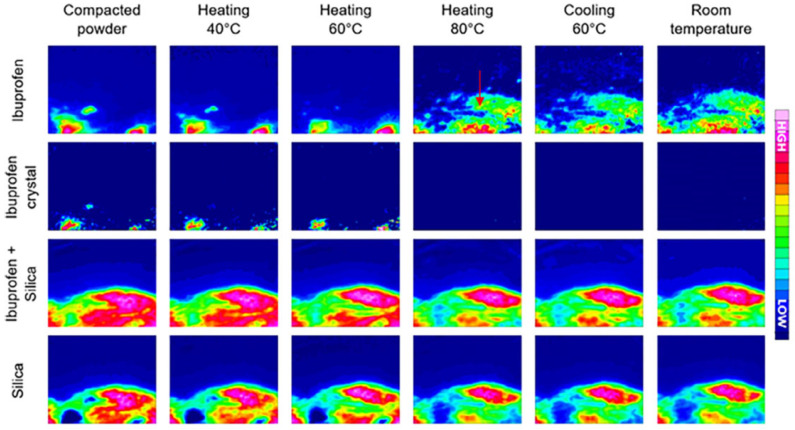
Hot-melt loading process followed by ATR-FTIR spectroscopic imaging. In the **first row**, ibuprofen in both amorphous and crystalline forms was detected based on the 1705 cm^−1^ peak. In the **second row**, crystalline ibuprofen in amorphous form was mapped based on the peak at 2922 cm^−1^. The **third row** presents the localisation of ibuprofen and silica combined; the **last row** shows the detection of silica by itself. Image size: 635 µm × 525 µm. Reprinted with permission [80].

In a paper by Lizonova et al., the loading process of ibuprofen into mesoporous silica microparticles via hot-melt loading (HML) was explored using ATR-FTIR spectroscopic imaging. The resulting HML samples were compared to a physical mixture (PM) of ibuprofen crystals and silica particles in the same ratio, and their drug release was studied in both powder and tablet form. In addition to FTIR spectroscopic imaging, differential scanning calorimetry and scanning electron microscopy were used to characterise the bulk properties of the HML silica particles and PM sample [80]. The hot-melt loading process was successfully followed by ATR-FTIR imaging (Figure 6), as the crystalline and amorphous ibuprofen could be clearly distinguished based on the absorption peak at 2922 cm^−1^, corresponding to CH_2_ vibrations in crystalline ibuprofen. After reaching the melting point of ibuprofen, the molten drug is redistributed to other areas within the silica microparticles through capillary forces of the silica pores. Furthermore, the chemical images demonstrate that once the drug is molten, the crystalline form of ibuprofen is no longer present. Additionally, after the sample is cooled down to room temperature, crystalline ibuprofen does not show up in the chemical images, demonstrating that the amorphous form of ibuprofen is stable in the silica particles and that the hot-melt loading method was effective [80]. This was followed by a dissolution experiment, which revealed the rapid release of ibuprofen in amorphous state. However, local super-saturation promoted the formation of ibuprofen crystals, both at the surface of the tablet as well as within cavities. When comparing the chemical images of the HML tablet to the PM tablet during dissolution, it can be concluded that drug release is much slower for the ibuprofen crystals in the PM tablet than for the amorphous ibuprofen in the HML tablet. In addition, it was observed that dissolved ibuprofen from the crystals in the PM tablet interacted with the surface of silica particles, leading to the adsorption of ibuprofen molecules on the silica surface [80].

### 5.2. Importance of the Dissolution Environment: pH and Ionic Strength

The dissolution behaviour of oral dosage forms greatly depends on the environment it is exposed to, including factors such as temperature, pH and ionic strength. Investigating dissolution and drug release under various environmental conditions is therefore essential, especially in the context of the physiological conditions of the gastrointestinal tract fluid. For example, the pH of the stomach is highly acidic, whereas the pH of the intestines ranges from neutral to basic [81]. Furthermore, the ionic strength of the fluid in the gastrointestinal (GI) tract varies significantly depending on fasted or fed state [82]. ATR-FTIR spectroscopy offers the possibility to study oral dosage forms under a range of different conditions and compare their dissolution behaviour, as demonstrated by the studies presented in this section.

Velasco et al. evaluated polymer hydrogels as a matrix system for ibuprofen release under different pH values, i.e., 2, 5 and 7.4 [81]. The hydrogels were designed to prevent crystallisation of ibuprofen, as this does not only affect the drug release profile, but may also cause damage to the stomach mucosal membrane. Homopolymers and copolymers of N-ethylmorpholine methacrylamide (EMA) and N,N-dimethylacrylamide (DMA) were used for the preparation of hydrogels. ATR-FTIR imaging recorded the stretching vibrations of the carbonyl groups in each of the components, present in the ester groups of ibuprofen, EMA and DMA homopolymers. Based on the chemical images that were generated, it was found that ibuprofen was released mostly in non-crystalline form at a pH of 7.4 for the DMA hydrogels. This was explained by the polar interactions between ibuprofen and the dimethyl acrylamide groups of DMA. However, at the lower pH values of 5 and 2, poly-DMA could not prevent crystallisation. Incorporating poly-EMA in the hydrogel did not result in crystallisation at pH 7.4 and pH 5. Expansion of the polymer matrix was observed while ibuprofen was released and no crystalline ibuprofen was detected. At pH 2, crystallisation of ibuprofen was observed at the edges of the matrix, where poly-EMA was only present in low concentrations due to expansion and subsequent dissolution. These macro ATR-FTIR spectroscopic images indicated that there is a minimum concentration at which poly-EMA should be present in order to prevent crystallisation, which happens through polar interactions between the carboxylic group in ibuprofen and the tertiary amines in poly-EMA [81].

**Figure 7:**
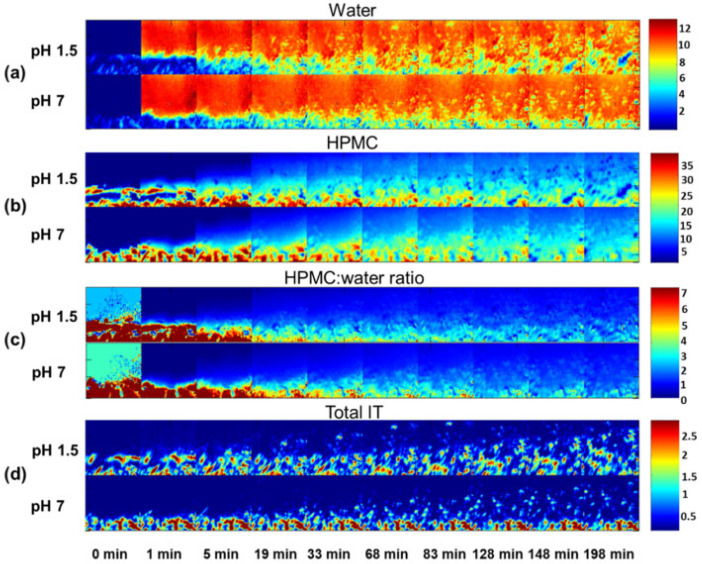
ATR-FTIR images showing (**a**) water distribution, (**b**) HPMC distribution, (**c**) HPMC/water peak area ratio and (**d**) IT distribution. The dissolution experiments in acidic (pH 1.5) and neutral (pH 7) medium at low ionic strength are presented. Image size: 640 µm × 640 µm. Reprinted with permission [83].

To understand the impact of pH and ionic strength on drug release rate from a hydrophilic matrix, ATR-FTIR imaging was employed to monitor tablets containing itraconazole (IT) and excipient hydroxypropyl methylcellulose (HPMC) [83]. ATR-FTIR imaging showed that gel layer formation and swelling were not influenced by pH but heavily depended on ionic strength of the hydrating medium in placebo tablets which only contained HPMC. Following this, HPMC tablets containing itraconazole were studied using a hydration medium of low ionic strength, while only varying the pH value, i.e., pH 1.5 and pH 7. The ATR-FTIR images of water, HPMC and HPMC/water are shown in Figure 7. Differences in swelling could be observed between the low and neutral pH conditions, as well as differences in IT distribution. At neutral pH, the drug is insoluble and does not easily dissolve or diffuse through the hydrated polymer matrix. IT therefore mainly resides in the core of the tablet, while the HPMC matrix swells, carrying some IT particles with it. At low pH, however, IT is much more mobile and is able to move with the diffusion front. Based on these observations, it could be concluded that polymer swelling is significantly different for the two pH values when IT is present in the tablet. To obtain a better understanding of the drug translocation phenomena taking place during swelling and expansion of the polymer matrix, IT particles in the FTIR images were characterized and tracked during their outward movement. This analysis confirmed that at lower pH, IT particles were more mobile and moved larger distances relative to the interface. This was explained by the higher drug solubility at low pH, which results in IT being able to dissolve out of the HPMC matrix, changing the porosity and expansion properties of the matrix during hydration. All in all, this study demonstrated the use of ATR-FTIR spectroscopic imaging for the analysis of drug release from hydrophilic matrices, specifically for poorly soluble drugs under different pH environments [83].

**Figure 8:**
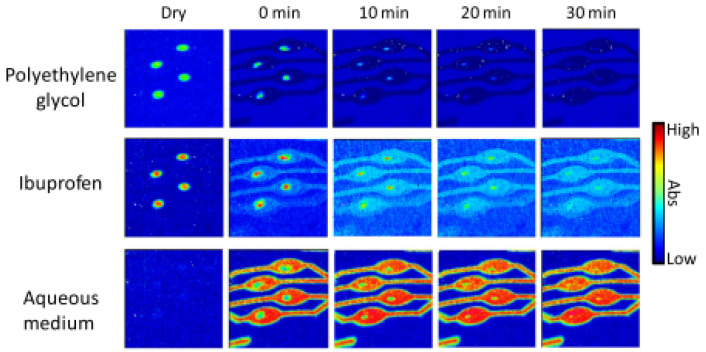
ATR-FTIR images showing the distribution of PEG, ibuprofen and the aqueous medium during the dissolution experiment of four ibuprofen/PEG (1:3 *w*/*w*) tablets. Image size: 11.5 × 8 mm^2^. Reprinted with permission [84].

A pioneering study by Ewing et al. developed a high-throughput method to analyse the drug release from micro-formulations in situ by using ATR-FTIR spectroscopic imaging in combination with polydimethylsiloxane (PDMS) microfluidic devices [84]. For this purpose, a model formulation of ibuprofen and polyethylene glycol (PEG) was studied under various pH conditions. The microfluidic devices were designed with multiple inlet and outlet channels, such that multiple micro-formulations could be studied at the same time. In addition, another device was designed to study dissolution behaviour using two different aqueous media, as well as a device to study mixing of dissolved drug with a medium of different pH. The authors demonstrated the flexibility of device fabrication and the versatility of combining microfluidics with chemical imaging. The chemical images that were obtained from the in situ dissolution experiments using ATR-FTIR spectroscopic imaging showed that at neutral pH, ibuprofen is completely released from the matrix formulation after 20 min. Accordingly, the ingress of water into the core of the formulation is observed over the same period. The results of this experiment are shown in Figure 8, which demonstrates the possibility of studying the dissolution of four formulations simultaneously. When this experiment was repeated using a low pH medium, i.e., with a pH 1 HCl solution, the dissolution behaviour changed. Both ibuprofen and PEG displayed limited dissolution and remained at the core of the formulation. In addition, no water ingress was observed. In order to understand the underlying cause, the ATR-FTIR spectra of this experiment were analysed. It was found that upon contact with the low pH medium, ibuprofen was converted from molecularly dispersed form to crystalline form. Since dissolution for crystalline ibuprofen is slower than for molecularly dispersed ibuprofen, slower drug release was observed. Furthermore, it seems to reduce water penetration, thereby potentially also decreasing the rate of dissolution of PEG.

**Figure 9:**
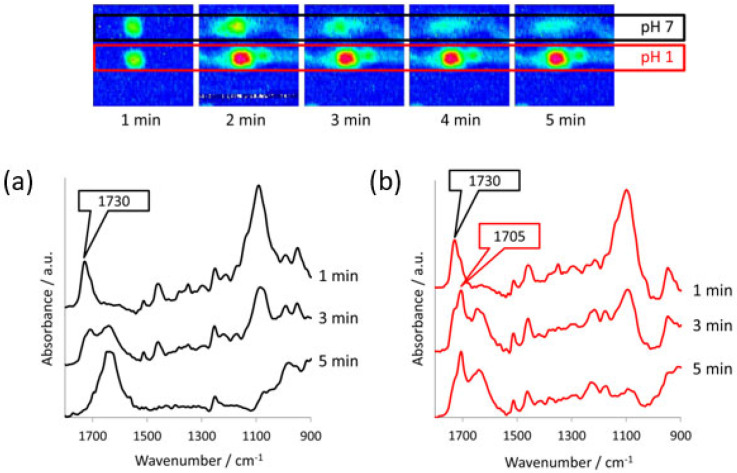
**Top**: ATR-FTIR images of the dissolution of two ibuprofen/PEG (1:3 *w*/*w*) tablets in separate channels with different pH medium, i.e., pH 7 and pH 1. Image size: 7.75 × 6 mm^2^. **Bottom**: ATR-FTIR spectra extracted from (**a**) the pH 7 chemical images and (**b**) the pH 1 chemical images. The peaks characteristic of crystalline ibuprofen (1705 cm^−1^) and molecularly dispersed ibuprofen (1730 cm^−1^) are indicated. Reprinted with permission [84].

The results described above were confirmed by studying the formulation under both pH environments simultaneously by using a microfluidic device accommodating two separate flow channels. In this setup, presented in Figure 9, dissolution with the pH 7 medium and pH 1 medium could be studied at the same time. Finally, the study investigated the crystallisation of sodium ibuprofen dissolved in a neutral solution upon mixing with a pH 1 HCl solution. The microfluidic device used for this study consisted of a T-junction where the two solutions mix, followed by a serpentine channel. This setup successfully showed recrystallisation of ibuprofen upon mixing with the acidic medium, which could be further confirmed by analysing the extracted spectra from the chemical images [84]. Most importantly, this study demonstrated the use of microfluidic devices in combination with ATR-FTIR spectroscopy imaging to study micro-formulations under various conditions. This may be particularly useful for high throughput screening purposes or in the case of costly formulations.

### 5.3. Multi-Layer and Multi-Drug Tablets

In addition to monolithic matrix systems, other complex drug delivery systems have been developed, including multi-layer and multi-drug solid oral dosage forms. Multi-drug therapies incorporate multiple drugs into a single dosage form, thereby improving patient compliance as they offer convenience, reduced dosing and cost savings. Multi-layer dosage forms allow for the improvement of drug release profiles by addressing problems such as burst effects and non-linear release, as well as achieving zero-order release kinetics, sequential release and sustained release. Furthermore, multi-layer dosage forms may be able to incorporate chemically incompatible components and minimise drug side effects [85,86,87].

In a study on bilayer tablets consisting of two excipients, microcrystalline cellulose (MCC) and glucose, and two model drugs, nicotinamide and buflomedil, ATR-FTIR imaging was successfully employed to observe the dissolution of each component and their distribution within the biphasic tablet [64]. Each half of the biphasic tablet contained one of the drugs and the two excipients, which were added in various weight percentages. The poorly soluble MCC was added to slow down the drug release rate, whereas glucose was added for its high solubility and ability to promote water penetration. The aim of this study was to better understand redistribution of components and the structural changes occurring in multi-layered tablets during tablet dissolution. Ultimately, an understanding of these processes would provide a basis for the development of new formulations in which drug release can be accurately controlled.

**Figure 10:**
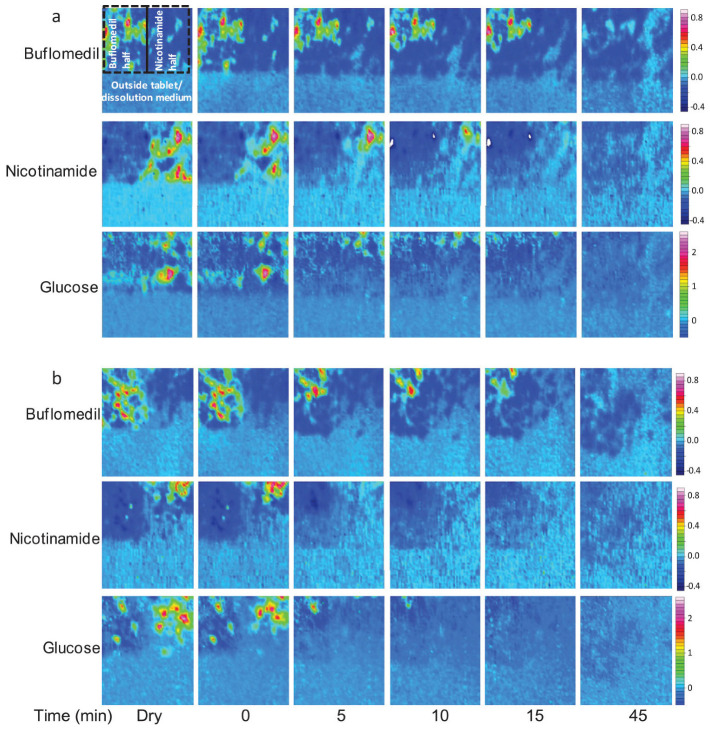
ATR-FTIR images showing the dissolution of (**a**) tablet A containing a low loading of glucose on the nicotinamide side, i.e., 10 wt% glucose, and (**b**) tablet B containing high loading of glucose on the nicotinamide side, i.e., 40 wt% glucose. Image size: 690 × 610 µm^2^. Adapted with permission [64].

For the biphasic tablet, two cases were compared in detail. That is, a tablet with high loading of glucose on the nicotinamide half and low loading on the buflomedil half (tablet B), such that any interference between the two tablet halves could be investigated. As a reference, another tablet containing a low weight percentage of glucose in both halves (tablet A) was studied under the same conditions. The ATR-FTIR images of both tablets during dissolution are shown in Figure 10. It was found that the half with high glucose loading dissolved rapidly as a result of increased water ingress. This led to an increase in the surface area of the half that is less soluble, and therefore improved the drug release rate for this section. Furthermore, two more formulations were investigated, containing HPMC and MCC as excipients and buflomedil as the model drug. Tablets were produced as (i) monolithic tablets and (ii) layered tablets with 100% HPMC as the barrier layer. These formulations were studied with UV-vis detection and ATR-FTIR spectroscopy, in order to investigate the effects of the constricted geometry of the flow cell used in a typical ATR-FTIR spectroscopic imaging setup. The constricted geometry of tablets in the flow cell was compared to the barrier-layered formulation, exhibiting zero-order release. It was found that only at relatively high flow rates did the dissolution profile resemble that of zero-order release formulations, attributed to the erosion of the gel layer [64].

In a more recent study by Lee et al., the dissolution behaviour of Fesoterodine fumarate (Fst) and Mirabegron (Mrb) was studied in a bilayer tablet where extended release was desired for both APIs [88]. Several techniques were implemented to characterize the dissolution behaviour of the two drugs in the bilayer tablet, including camera imaging, SEM, swelling and erosion studies, USP dissolution testing and ATR-FTIR spectroscopic imaging. At three time points during dissolution, bilayer tablets were freeze-dried and their cross-sections analysed using ATR-FTIR spectroscopic imaging. Using ImageJ, a 3D image could be constructed from the chemical images, such that the distribution of both Fst and Mrb could be observed in each layer. The chemical images and 3D representations showed the drug transfer for both Fst and Mrb. First, it was observed that the amount of Fst in the original Fst monolayer slowly decreased, while the amount of Fst in the Mrb monolayer gradually increased over time. This could be explained by swelling of the Fst monolayer, transferring the drug to the Mrb layer and delaying Fst release. However, this did not significantly affect the overall dissolution profile, compared to Fst drug release in monolayer tablets. For Mrb, the results were considerably different. The amount of Mrb in the original Mrb monolayer remained roughly the same during dissolution as Mrb is mainly released from the tablet through erosion. Furthermore, only a small amount of Mrb migrated into the Fst layer and a clogging effect of the Fst layer was observed. This resulted in a decreased dissolution profile as compared to the dissolution profile of the monolayer tablet. The use of ATR-FTIR spectroscopic imaging in this study revealed the amount of drug transfer from one layer to the other and vice versa in a dual-release bilayer-tablet system [88]. This paper demonstrates that, with more complex drug delivery systems under development, chemical imaging will become essential in the characterisation of the drug release mechanisms underlying dissolution profiles obtained via USP standard testing.

**Figure 11:**
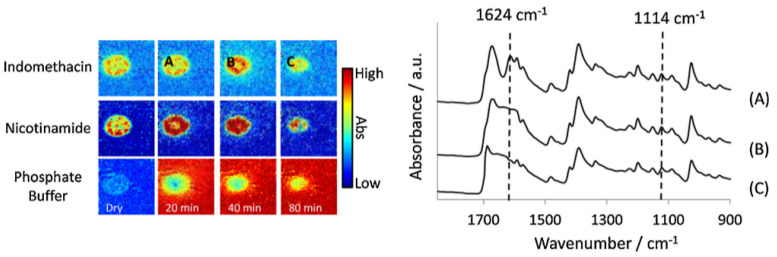
**Left**: ATR-FTIR images of the dissolution of a compacted tablet containing indomethacin and nicotinamide. The distribution of indomethacin, nicotinamide and phosphate buffer are shown over time; the letters **A**, **B** and **C** indicate the locations from which spectra at different time points were extracted. Image size: 7.75 × 6.05 mm^2^. **Right**: Extracted spectra from locations **A**, **B** and **C**. The peaks at 1624 cm^−1^ and 1114 cm^−1^ are representative of the co-complex of the two drugs, which was present at all three time points. Reprinted with permission [89].

In another study by Ewing et al., the dissolution behaviour of indomethacin was studied in compacted tablets with different types of excipients [89]. These included urea and mannitol, as well as a second drug, i.e., nicotinamide, at different weight percentages. The aim of the study was to understand the interactions between the two components—drug and carrier—and how drug release of a poorly soluble drug (indomethacin) could potentially be improved by adding a more soluble drug (nicotinamide) to the dosage form. The formulations were produced via the hot-melt method in order for indomethacin to be in amorphous form, and subsequently analysed through ATR-FTIR spectroscopic imaging, combined with UV detection. From the ATR-FTIR spectra measured after preparation of the tablet, it was concluded that nicotinamide formed hydrogen bonds with indomethacin. If these interactions would be maintained during dissolution, nicotinamide could be a carrier for indomethacin and improve its drug release. The extracted spectra that were obtained from the ATR-FTIR images, presented in Figure 11, showed that throughout the dissolution experiment, the interactions between the two components are maintained, and that nicotinamide acts as a carrier. In addition, the ATR-FTIR images showed that lower indomethacin loading resulted in better dissolution. This could be indicative of a second release mechanism, that is, the disintegration of the tablet matrix as a result of nicotinamide dissolving quickly, causing a larger surface area of indomethacin to be exposed to the medium, ultimately resulting in faster drug release.

The release of indomethacin in seven tablets of different drug loadings was further investigated based on the UV detection data. Unexpectedly, studying the release rate of tablets with ratios of indomethacin–nicotinamide of 50:50 and 75:25 showed similar amounts of drug released at the end of the dissolution test. The different intermolecular interactions, confirmed by changes in the extracted spectra, offer an explanation. The carbonyl group in indomethacin and aromatic functional group in nicotinamide could interact through H-bonding. However, indomethacin could also bond with other indomethacin molecules and form a dimer (γ-form). When there is proportionally more indomethacin, there is more competition for this interaction to occur. Furthermore, in tablets with higher loading of indomethacin, once nicotinamide has dissolved, more of the area of indomethacin is exposed to the medium and zero-order release can be observed. Higher drug loading does not always lead to more drug being released. From the resulting dissolution profiles it was clear that the ratio and drug loading are of great importance and determine the underlying mechanisms of drug release in the multi-drug system. This was explained by the intermolecular interactions at play in a multi-drug system and how these may differ depending on the loadings of both drugs present in the formulation. Despite the complexity of multi-layered and multi-drug tablets, these types of dosage forms may open up opportunities for improved release profiles and more efficient oral dosage forms [89].

### 5.4. Pharmaceutical Films

Hifumi et al. was the first to focus on polymeric pharmaceutical films using macro ATR-FTIR spectroscopic imaging and their possibility to be an alternative for capsules and tablets for patients encountering difficulty swallowing [90]. This type of dosage form was introduced in the late 1970s and has now become a standard in vitamins and personal care products. Most of the oral films on the market are formulated to dissolve completely in one minute after exposure to saliva, without drinking or chewing [91].

As a therapeutic dosage form, oral films have recently gained interest for pharmaceutical development. These films are mostly prepared via solution and dispersion states, and their physicochemical properties should therefore be carefully analysed throughout drying and dissolution, ensuring the safety and efficacy of this oral dosage form. In a study by Hifumi et al., macro ATR-FTIR spectroscopic imaging was presented as a useful analytical tool for the analysis of polymer-based pharmaceutical films during drying and dissolution [90]. Ibuprofen was used as a model drug for the polymeric films, with two types of polymer under investigation: HPMC to study immediate release and polyvinylpyrrolidone (PVP) to study extended release. Furthermore, the pH environment was varied, as well as the hydrophobicity of the films, through the addition of sodium carbonate or PEG 4000. Using macro ATR-FTIR spectroscopic imaging, it was possible to characterize the polymeric films, including the distribution of components within the different formulations, dissolution behaviour, and drug–polymer interactions. First, the drying process of the pharmaceutical film, which was prepared via a solvent casting methods, was studied. Different from tablet compaction, the drying process may result in chemical instability and undesirable drug–polymer interactions. The ATR-FTIR images followed the distribution of components in the film over time during drying. From the extracted spectra, it was found that the carbonyl stretching band underwent a shift to a higher wavenumber, which was attributed to hydrogen bonding between the C=O group of ibuprofen sodium salt and the O-H group of glycerol and HPMC. Overall, it was found that the ibuprofen sodium salt in the film was stable during the drying process. Secondly, the dissolution behaviour of HPMC- and PVP-based films was studied. Without a pH modifier or hydrophobicity modifier, the HPMC-based film exhibited faster dissolution, due to its hydrophilic character, compared to the more hydrophobic PVP. However, for the PVP-based film no crystalline ibuprofen was observed, whereas for the HPMC-based film this was the case. By extracting average spectra from areas of interest in the chemical images, it was found that upon dissolution of the film, the hydrogen bonding between the ibuprofen sodium salt and the HPMC and glycerol, which was characterized during spectral analysis of the drying process, was lost after water was absorbed into the film. Further experiments showed that addition of a pH modifier can increase the water ingress into the tablet, minimize the effect of the polymer forming a gel and result in no precipitation being observed [90].

### 5.5. Combined Use of Imaging Techniques for Drug Dissolution Studies

ATR-FTIR spectroscopic imaging provides both spatial and chemical information on a sample and can therefore be used as a stand-alone technique in drug dissolution studies, as demonstrated by the studies discussed above. However, when combined with other imaging technique such as magnetic resonance imaging [69] and Raman spectroscopic mapping [92], a more detailed understanding of the formulation and its dissolution behaviour can be achieved. For example, one of the potential limitations of ATR-FTIR spectroscopic imaging is the requirement for close contact between the sample and IRE surface, meaning that the tablet needs to be pressed down onto the ATR crystal and the dissolution medium reaches the sides of the tablet.

Puncochova et al. was the first to combine ATR-FTIR imaging and MRI to study the dissolution of a poorly soluble drug, i.e., aprepitant, formulated as solid dispersion with three different polymers, i.e., Soluplus, PVP and HPMC. Based on the ATR-FTIR and MRI images, the rate of water penetration and the rate of polymer erosion could be determined and compared for tablets containing Soluplus, PVP or HPMC. Qualitatively, the results obtained from ATR-FTIR imaging were the same as those obtained from MRI. That is, comparing the water penetration rate between different tablets resulted in the same conclusion. However, quantitatively, the results were different, i.e., the penetration rates found from ATR-FTIR imaging were significantly lower than those found from MRI. This could be explained by the tablet being physically sandwiched between the ATR crystal and the flow cell top plate. Nonetheless, ATR-FTIR imaging was able to identify structural changes in the drug corresponding to crystallisation of aprepitant in the PVP-based tablets as a result of local supersaturation and subsequent precipitation of the drug in absence of a gel layer. The MRI data, on the other hand, revealed the rate-limiting step in the dissolution process, which was found to be the diffusion of aprepitant through the gel layer [69].

**Figure 12:**
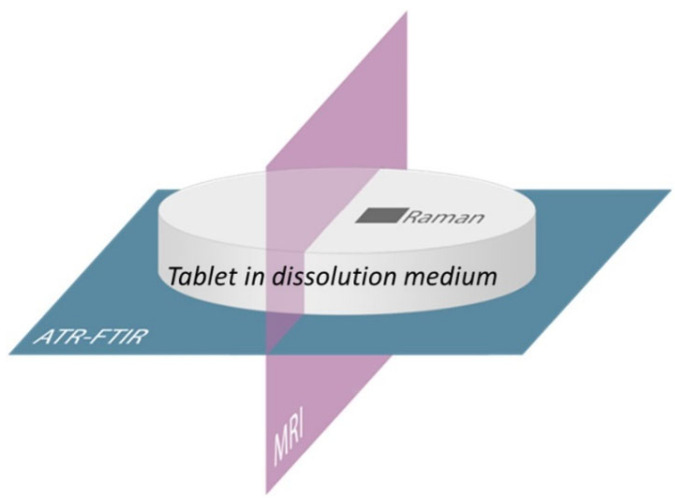
Schematic of combined imaging approach using ATR-FTIR imaging, Raman mapping and MRI. Reprinted with permission [6].

In a second study by the same author, ATR-FTIR imaging, confocal Raman mapping and MRI were combined for the first time to study amorphous solid dispersions containing two polymers, Soluplus and PVP, and the same drug, aprepitant. Here, the aim was to find the most suitable ratio of Soluplus–PVP in the mixed polymer matrix for fast dissolution while at the same time preventing drug crystallisation. Each of the imaging techniques provided different spatial and chemical information on the studied sample, and as such, they complemented each other [6]. Figure 12 shows the imaging positions of each of the three techniques applied in the study.

MRI, ATR-FTIR imaging and Raman mapping confirmed that tablets with ratio Soluplus–PVP of 1:1 were the most suitable. In that ratio, solubilizer Soluplus and dissolution enhancer PVP both contributed to the enhancement of the dissolution rate while at the same time preventing crystallisation. MRI gave insight into the rate of water penetration and was able to observe structural changes in the gel layer, including swelling and erosion. In addition, crystallisation could be detected using MRI based on a solid phase forming in the gel layer. Raman mapping and FTIR imaging were able to detect crystallisation based on structural changes in the drug from amorphous to crystalline state, which appear in their respective spectra. Specifically, ATR-FTIR spectroscopic imaging revealed the distribution of individual components, along with their structural changes, concentration profiles and diffusion rates during dissolution. Raman mapping was able to provide information on the local composition and structural changes at the free tablet surface in contact with the dissolution medium. Altogether, these three imaging techniques were successfully combined to optimize the mixed polymer matrix for enhanced dissolution of the API. Additionally, this paper presents a methodology for formulation development using MRI, ATR-FTIR imaging and Raman mapping [6].

### 5.6. Modelling Tablet Dissolution and Drug Release

Using mathematical models, tablet dissolution and drug release can be simulated and predicted. One way to develop a model for tablet dissolution is to fit experimental data using neural networks, which is a top-down data-driven approach. On the other hand, a model can be developed bottom-up based on the physical properties of the tablet components and fundamental principles of mass transfer. Other factors that should be taken into account when developing a model for tablet dissolution are the homogeneous/heterogenous dispersion of components, phenomena related to swelling, and multi-layer tablet compositions. ATR-FTIR spectroscopy and spectroscopic imaging have shown to be useful tools for finding physical properties through designed experiments and comparing model predictions to experimental data. Modelling may be useful to explore the design space of a formulation and understand the underlying mechanisms of drug release. In addition, a model can be used to optimise formulations or evaluate their stability through sensitivity analyses [6,93].

**Figure 13:**
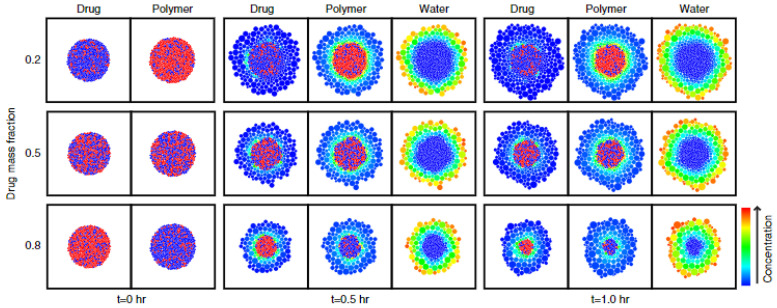
The distribution of drug, polymer and water is presented for tablets of different drug loadings, i.e., 20%, 50% and 80%, for a time period of 1.0 h. Swelling is represented by swelling particles and concentration is represented through the colour scaling. Reprinted with permission [93].

Kimber et al. applied the discrete element method (DEM), in which the dosage form is divided into units of discrete mass, so-called DEM particles, to develop a model for the dissolution behaviour and drug release from swelling tablets [94]. Dissolution behaviour and drug release were further investigated by performing parametric studies on some of the most important parameters for drug release, including the distribution of drug and polymer, the maximum swelling ratio and the diffusivity of the drug through polymer [93,94]. Figure 13 shows the concentration distribution of different components within a swelling tablet at different drug loadings resulting from the DEM-based model. In this case, drug and polymer were heterogeneously dispersed.

**Figure 14:**
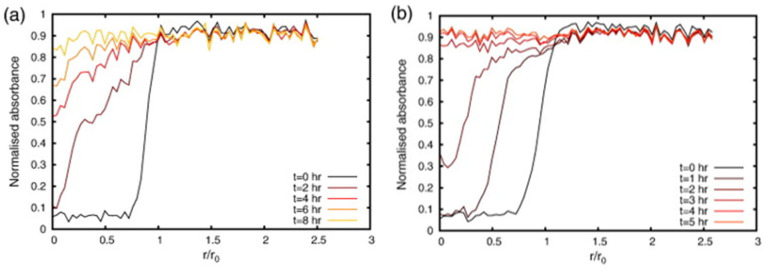
Absorbance profiles of water for the (**a**) 10% *w*/*w* nicotinamide tablet and (**b**) 60% *w*/*w* nicotinamide tablet. The normalised absorbance of water measured along the radius of the tablet is plotted for five time points during dissolution. Reprinted with permission [93].

The model was compared to experimental data obtained from ATR-FTIR spectroscopic imaging and UV-vis spectrophotometry. The dissolution and drug release of two dosage forms of different drug loads, that is, 10% *w*/*w* and 60% *w*/*w* nicotinamide tablets with HPMC as the excipient, were studied in a flow cell setup. The ATR-FTIR images were used to obtain absorbance profiles for water, HPMC and nicotinamide over time and over the tablet radius. Figure 14 shows the absorbance profiles of water at five different time points during dissolution of the (a) 10% *w*/*w* and (b) 60% *w*/*w* nicotinamide tablet.

The model was optimized based on the experimental data and the resulting drug release curves compared. Although both curves were fitted relatively well, drug release time was over-predicted for the 10% *w*/*w* tablet and under-predicted for the 60% *w*/*w* tablet, indicating that the model needed further improvement. Specifically, a difference in tablet porosity, related to tablet compaction properties, was mentioned as one of the factors that should be taken into account [93].

**Figure 15:**
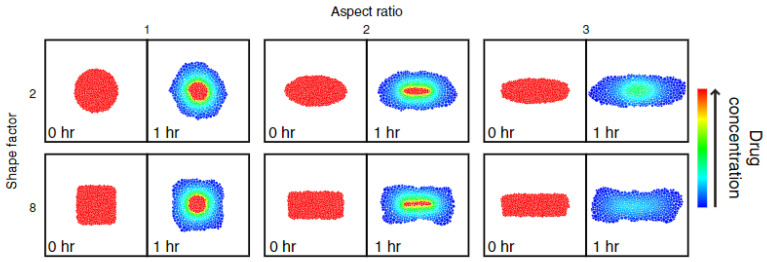
The drug concentration distribution in tablets of different aspect ratio and shape is presented for time point t = 0 h. and t = 1 h. of drug release. Reprinted with permission [95].

Building onto the developed DEM model, Kimber et al. included several other factors to be able to investigate different dosage form structures and shapes, as well as implement boundary conditions. Figure 15 shows the results of changing the shape and aspect ratio of the tablet and the effect on drug concentration during dissolution. Three parametric studies were conducted, which included (i) tablet shape and aspect ratio, (ii) shell thickness and polymer properties, and finally (iii) the sink conditions surrounding the tablet [95]. In this work, the model was validated based on a reference model for drug release from a swelling tablet, rather than compared to experimental data.

## 6. Conclusions and Outlook

In this review, a variety of studies have been presented which employed ATR-FTIR spectroscopic imaging for the investigation of dissolution behaviour and drug release mechanisms of solid oral dosage forms. These included the comparison of different excipients, component ratios and medium properties, as well as formulation and fabrication methods. ATR-FTIR images were able to reveal physical processes such as swelling and drug translocation, but also chemical processes including crystallisation and drug–polymer interactions. The combination of spatial and chemical information that is obtained through ATR-FTIR spectroscopic imaging allows for a much deeper understanding of dissolution behaviour compared to the standard USP dissolution tests, and the technique therefore has great potential for the wide-scale implementation in the pharmaceutical industry. Several key characteristics, including the ease of sample preparation, the unrestricted sample thickness and the flexibility of the flow cell setup, make ATR-FTIR spectroscopic imaging a particularly powerful analytical tool for investigating tablet dissolution and optimizing current formulations. When combined with other imaging techniques, such as MRI or Raman imaging, a more thorough understanding of the sample and its dissolution characteristics can be achieved. Furthermore, the combination of FTIR spectroscopic imaging and modelling approaches was highlighted. Tablet dissolution models that are able to simulate the dissolution process and predict dissolution kinetics under several different conditions may significantly reduce the amount of time spent on experimental work as they allow for design space analysis and parametric studies. Likewise, high-throughput measurement setups increase efficiency as multiple samples can be studied simultaneously.

There is a significant trend of using quantum cascade lasers (QCL) as a source of infrared radiation [46,47]. These are discrete-frequency tuneable lasers, which generate mid-infrared radiation several orders of magnitude brighter than conventional infrared sources in infrared spectroscopy. This provides a very high signal-to-noise ratio compared to FTIR spectroscopic imaging. The other advantages include much faster mapping and absence of fluorescence when compared to Raman mapping, and very fast measurements compared to FTIR imaging systems. However, while QCL is indeed a powerful tool, it remains a discrete-frequency infrared system. Therefore, it may not always be suitable for specialised studies, such as revealing and understanding the mechanisms of drug release during tablet dissolution. Nevertheless, for routine studies, such as analytical quality control for well-characterized systems and continuous manufacturing of pharmaceuticals, it promises to be a very fast and reliable tool [96].

## Figures and Tables

**Table 1:** Overview of IRE properties [63].

IRE Material	Refractive Index ^1^	Depth of Penetration ^2^	Hardness (kg/mm^2^)	pH Range
Diamond	2.4	2.0	5700	1–14
Zinc selenide	2.4	2.0	120	5–9
Germanium	4.0	0.66	550	1–14
Silicon	3.4	2.0	1150	1–12

^1^ At 1000 cm^−1^. ^2^ At 1000 cm^−1^ for a sample with refractive index of 1.5 and a 45° angle of incidence.

## Data Availability

Not applicable.
